# Opening Address Delivered before the Associated Alumni of American Dental Colleges, at a Meeting Held at the Baltimore College, March 2, 1854

**Published:** 1854-04

**Authors:** Elisha Townsend


### ARTICLE V.

*Opening Address delivered before the* ASSOCIATED ALUMNI OF AMERICAN DENTAL COLLEGES, *at a Meeting held at the* BALTIMORE COLLEGE, *March* 2, 1854. By ELISHA TOWNSEND, D. D. S., M. D.

GENTLEMEN:

Your association is so happily adapted to those exigencies of the profession which it is intended to meet and answer, it is so clear an index of our rapid progress, and so conclusive a pledge for the early fulfilment of our highest hopes, that I can find no words fittingly to express my apprehension of it.

It seems to me quite impossible for *you* to understand the feelings with which the men, whom I am understood to represent here to-day, look upon the organization of the Society of the Alumni of the Dental Colleges of America.

It is an *era* to *you*, from which you will measure the onward march of your own development and achievements for the profession—it is an *epoch* to us, that marks an advanced stage of attainment, a resting place in the history of our labor, a new-year's day in our calendar, bounding one complete cycle of progress, and opening another in perspective. To *you* an expectation is born, to *us* a hope is fulfilled, an aspiration is realized, a task is accomplished, a long wished, toil-won eminence is reached.

This, which is your present and point of departure, is that future of our early hopes to which our eyes have looked and our steps have tended, as to an accepted terminus of our fond ambition, and the feeling is strong upon us to sit down now and rehearse the history of the past, its struggles, and its triumphs, and live over again in the accomplishment the pleasures which it so often yielded us in the anticipations of an assuring faith.

Our eyes would fondly linger by the way, tracing on memory's map the devious, difficult, but dete mined pathway of that progress which your birth-day crowns and celebrates; but your gaze is turned with ardor into the prospect that opens up before you, and we must turn with you, forgetting the things which are behind, and reaching forth unto those things which are before, and press together toward the mark for the prize of our high calling in a very noble profession. When I speak of this day, distinguished by its grand event, as "an high day," a holyday; I mean not that the repose it brings to our solicitude, and the refreshment it ministers to our wonted toil, may rightly diminish devotion to our duty, or afford a furlough from its service, but, in my conception, the occasion has the character of a festival, affording a happy hour of liberated thought and feeling, strongly tempting the fancy to wander at will in the track familiar to the footsteps of those who have made the journey which brings us to this hour of happy consummation. But I am recalled from those pleasant passages of the beaten pathway to stand with you upon the high ground that we have reached, and look around and before us, to leave the route of recollection for the survey of the field we occupy, and the prospect that opens beyond it.

Where stand we, and what lies within the circuit of our vision? One glance at the retrospect, and then let us look steadily at our position and our duties. Twenty-five years ago, dentistry was practiced as a secret art. It neither was, nor pretended to be, a liberal, any more than a learned, profession. Then two or three dentists sufficed for the practice of Boston, an equal number for Philadelphia, and in the same proportion, for New York, and the other Atlantic cities, and the great west and south were served, where they were served at all, by itinerants, whose visits were occasional and accidental, and their benefits, most likely, of much the same character.

A few illustrious instances there were of men of mark, in those early days, like the heroes and prophets of a barbarous age, but their very distinction exposes the poverty of the times in which they flourished. There are no giants in civilization, and geniuses are comparatively rare when culture is general, and the common standard is high. "A commonwealth of kings" is a better condition than a community of churls, with a prince or two reigning above them, out of the reach of their capacity for anything but wondering worship. Now, distinction is better leveled, and more broadly distributed. Not less than three thousand practitioners occupy the places of the three scores of a quarter of a century ago, and the average capacity of the mass is in at least an equal ratio with the numbers—as much better as they are more numerous.

This for the general comparison and the census of the subject. The specific and distinguishing features have still more value in the estimate.

Fifteen years ago, the first regular dental college in this country, and the world, was established in this city, and already this noble pioneer in classic education is followed by three others favorably situated for the usefulness they aim at, and equally devoted to the best interests of the profession. Fourteen years ago, the American Society of Dental Surgeons was formed, which has been followed by numerous local institutions, and to-day you have founded the new order of Associated Dental Alumni of the nation!

In the mean time, the text books and treatises of the science have grown into an encyclopedia, and our periodicals cover the ground, and supply the current wants, of the profession. This, it seems to me, completes the circuit of institutional agencies, and describes the outline of instrumentalities by which it is to accomplish its educational functions, and achieve its destiny as an independent branch of the healing art.

What a history is this to lie within the compass of half an ordinary life time!

It seems but as yesterday when there were not so many even of the poorest pretenders to our art, as there are to-day accomplished graduates of our colleges!

Judge, then, with what gladness we meet you here, in the character and for the purpose that has assembled you. Imagine the exultation that we feel in these first ripe fruits of the harvest, on which our hopes have hung, and rejoice with us in the confidence which such success inspires, of all we have yet to wish for.

To this point we have arrived. The principles of our art have taken the form of a systematic science, its practice has been brought within rule, a literature, adequate to the exigency, has been established, formal education in its highest style of efficiency is fairly inaugurated, a fraternity of legitimate practitioners is acknowledged, a standard of worth, with accrediting designations, is set up and accepted, all the interests of the profession are embraced and conducted by the highest idea in it, and the methods and means of defence and development are richly possessed, and sufficiently active.

So, the transition age is passed, and the conditions and duties of a new era are upon us; upon *us*, I say, for the men who have lived in these hopes, and labored to these ends, are neither old enough, nor indifferent enough yet, to surrender their interests, or abandon their duty to the times that still lie before and rise above us.

Let us look over into this land of promise, and survey briefly the route of progress into it.

The first measure essential to the advance is, efficient professional organization, with its policy adjusted in every direction to the cultivation of the science, and to the regulation of the conduct of the profession.

This, in one of its departments, you have happily instituted. Your scheme contemplates the gathering into one associated fraternity the graduates of the schools, that you may at once provide for all the interests of your order, and avail yourselves of the help that there is in association for individual advancement, and of its power in forming an authoritative professional sentiment, and through its agency, raising public opinion into a supporting conformity.

You cannot expect too much from such an organization, for it is capable of all the good and more, it may be, than the most sanguine among you hope for; but it must not be forgotten, also, that it may fall below and disappoint even the most moderate calculations that any man who enters it may entertain.

A society may be anything in results, from an honorable association, down to a selfish conspiracy; a great good or a great evil; or, it may be a mere nothing—and again, it may be all right in its principles, and yet wholly inefficient in practice.

For what general objects, and in what spirit, should the graduates of dental surgery gather themselves into one great family? In the first place, you have yourselves taken the regular steps for attaining a thorough professional education, you are the first instances and witnesses of the system so lately introduced among us, and it behooves you to vindicate this new movement by demonstrating all those benefits, the expectation of which induced its adoption. You are to show that you have been, not only more regularly, but better, educated than you could have been without the advantages of college training. One of the best proofs of this point which you can give to the world is, an increased and intensified desire for all learning, scientific and practical, that holds any effective relation to your profession. If you make your association serve for mutual help in inquiry and attainment, however slight the actual gain, you will have justified the system of collegiate education triumphantly. Whatever leads to such an effort, and induces such a spirit, fulfils the highest promise of an educational enterprize.

A method of study which teaches its pupils only to think, and inspires a hungering and thirsting for legitimate knowledge, is worthy of all acceptation, even if, directly and immediately, it imparted not a single preceptive or practical truth, if that were not in the nature of the thing, quite impossible. Education, indeed, is not so much that which is put into the mind, as it is that which is evoked or brought out of it. It is not the transfer of the goods of a storehouse of learning to passive recipients, but it is the power of the keys by which aroused intellect may enter all repositories of treasure, and for itself take available possession. A surgeon may show his student how to reduce a fracture, or a dentist may exhibit the method of treating a diseased tooth, but the successful instructor is he who prepares his pupil to grapple with practice, either routine and familiar, or new and unknown, in the spirit, and with the power of a discoverer. Let your association only indicate an insatiate desire to press forward in professional attainment, and the collegiate system will stand warranted and honored in the judgment of all men.

While you thus exhibit the utility, and carry the issues of the system forward toward their ultimate aim, you are also concerned to assert its dignity and maintain it, not in any spirit of selfishness, exclusiveness, or diplomatic vanity, but to effect a wholesome and necessary reform of the commonly low standard of professional competency.

The honor of the profession is in a distinguished degree in your keeping. First, to be promoted by your own real eminence in all its functions, and next, by the guard you set over its character in the requirements you make as conditions of professional recognition. Quackery is to be discredited as decidedly as legitimate qualification is to be respected and supported. This is difficult and delicate ground, but cannot be dangerous to enlightened and liberal men. Remembering that the very preceptors whom you most honor, were in the profession before a dental college was in existence, you cannot commit the mistake of holding a diploma to be the sole and exclusive evidence of respectability, though it may very properly be the condition of membership in your special association. On the other hand—to the extent that your opinion and influence can affect the system of study of those about to enter the profession, if you are convinced that the collegiate course is to us what it is to the other branches of general medicine, you will not fail to lend all your influence in its favor; and in like degree, and for the like generous and just reasons, discourage and discountenance the usage that undervalues and evades it. The idea and effort which have resulted in the successful establishment of the collegiate method depend upon you for the demonstration of their worth. In scripture language, "wisdom is justified of her children." This duty rests upon you. This responsibility you have incurred, and you can discharge it only by an enlightened devotion to the interests of your own order, and an unselfish exertion of all the power you possess to discharge its duties to the world.

Medical societies, prompted by an absorbing zeal and pride of caste, are liable to convert themselves into close corporations, to the neglect of those generous obligations which they owe to the world outside of their limits. The regulation of their fee-bill, declamatory denunciation of quackery, fastidiousness in association, and an exacting system of etiquette among themselves, may easily receive more than due attention; and when these are the sole or principal drift of the organization, nobody but the members are concerned in them, and no one else will long respect them.

Giving these things their due and necessary place among your objects, you will nevertheless hold them properly subordinate to the greater aims of your fraternity. The interests of a liberal profession are intrinsically worthy of high and earnest consideration, while kept in harmony with its duties; and they are doubly so, for the reason that its prosperity is an essential condition of its capability for the most generous uses of its existence; but these highest purposes of its being, as they are the ultimate, so they should be the governing aims of all its conduct.

Let us look for a moment at the capabilities of your association to forward its noblest intentions. You have finished the studies of your pupilage, and some of you have already entered upon responsible practice. You are in that stage of professional life that brings the principles of your art to the test of personal experience. The available truth of your teaching, and your own clearness of apprehension, and thoroughness of attainment, are put under trial upon the testimony of practical facts, and before the jury of the country. You are just in the position to need counsel, and in your case it is eminently true that in a multitude of counsellors there is safety. Your society, if nothing else, is a hopeful refuge for the earnest solicitudes of your position, and, it is capable of meeting your necessities in a multitude of particulars.

In the first place, there is a natural tendency to relax theoretical study amid the anxieties and engagements of an opening practice. It is not felt to be a direct means of securing the early success that, for so many good reasons, is desirable. There are a thousand seductive influences at work, whose tendency is to abate the enthusiasm of professional study, and crowd it out of place. You do well to provide yourselves with counter influences against these diversions of the intellect. The most ardent of you will be helped to effort by the obligation to conventional duties incident to membership. For every one of you some service may be appointed, which will demand the assiduity, and keep up the habit of studious inquiry. You will be living in the presence of your peers, and will feel all the force that there is in laudable ambition to impel, and in respectful but critical expectation to invite you, to creditable exertion.

The regularly recurring assemblages of the society, like mile stones, will indicate the progress of each mind, or like tombstones, mark the loss of growth and vitality, and will press the knowledge and consciousness of his condition upon him, and thus act as provocatives to all that is possible in him. For mere disciplinary purposes, every man of you will find his membership a means of saving grace against all temptations to indolence and delinquency. Whoever knows his own nature best, is most earnest in guarding and strengthening his convictions of duty by every corrective that he can judiciously provide. Task yourselves to your best performances, and give the pains and penalties of opinion the best opportunity for compelling you to obedience. Not one man in a thousand gets all his resources into their best activity without the help of other incitements and responsibilities than those that are entirely within his own government, and liable, therefore, to be dispensed with, under the excuses which only too readily present themselves.

When a man is a whole society himself—president, recording secretary, and treasurer—the pledges he makes to it are far from immutable. When pleasure, profit or idleness interfere, he has nothing to do but call the society together, and postpone, modify, or abolish the pledge, by a unanimous vote. Better let somebody else have a word to say in such meetings for the transaction of business. Better have the exactions of less complying parties to meet, than thus to be at the disposal of your own caprices. I speak from the experience of one who is conscious of doing his best work of every kind under such compulsions as he would, many a time and oft, have dodged, if the veto power had not been lodged in other hands and heads than those which had the work to do. I need not make more explicit confessions, for I don't want to be personal, but let me whisper in your ears, in a voice that gathers its strength and volume from the cumulative convictions of long years of struggle, and from the earnestness of anxious self-inspection, that the best thing you can do for the usefulness, honor and happiness of your professional lives, is to go into the collar and traces of duty resolutely, and take the checks and whip kindly, in the car of professional progress. I have but little hope of that man paying his debt, who refuses to give his note of hand for it with interest, when it has a long time to run; and I have as little hope of a zealous discharge of duty by that man who does not, at the outset of his career, bind himself to its performance. It is too much like marrying your sweetheart without the vow and the ceremony—too much like entering upon an office without giving the qualifying inaugural oath or affirmation which owns the trust and promises fidelity.

The yoke of a generous public service may not always sit lightly upon a limber neck; but principle and policy alike command its unequivocal and unflinching assumption; *you* are fairly hitched up, see that you run your course under a tight rein and a stiff trace.

But there are other necessities for such associations besides those that act chiefly as incentives to profitable self-discipline. The studies and teachings of pupilage are necessarily restricted, mainly to the elementary learning of the art; but all around the borders and above the level of this fountain of learning are tributary streams of science that must be invited into it by works of self-improvement necessary to keep the reservoir fresh, full, and flowing. The collateral branches of learning which run along the main channel must be explored and drained of their refreshing riches. In the exercises of your society the commonplace and familiar will of course be avoided, and the contributions made and received, will be from those sources that demand a multitude of minds in their multiform variety of fitness, and are just of the character that no single man can accomplish for himself in any tolerable completeness, and which no tolerably complete professional character can spare.

Among a hundred ambitious cultivators of science, not a few will furnish indispensable help to their compatriots; all will reap the benefit, and, what is best of all, the worthiest will be the standard and the stimulus of all. The excellence of such institutions is in their power to induce a noble emulation, and provoke a generous zeal, whose tendency is to level up the whole mass toward the highest in rank of its constituents.

The *esprit du corps* feeds the fire in every bosom, and the habit of effort, while it keeps the aim high, preserves the youthfulness and energy of ambition fresh and glowing.

You will go back to your every-day engagements from these conventions with the life of the whole association glowing in you, and will distribute its impulses all around you. Under these influences that study, which those who study much know best is never done, will happily never stop undone and unendeavored; and the rust of sloth will not be allowed to steal over and eat out the life of your young energies.

I can but hint at those ancillary arts and sciences which may be made richly to contribute to the general improvement of your society, through the diversified capabilities of its large membership; but I will hope that such a hint may sufficiently serve its purpose.

The entire range of general medicine lies within the purview of your own professienal studies. No man engaged in any one branch of curative practice can cultivate every kindred branch to the entire available value of all the treasures which it holds in waiting or his use. No book, or number of books, and no course of professional lectures upon our speciality, comprehends the full breadth and depth of these vast reserves of kindred knowledge. Such completeness, perhaps, cannot be affirmed, as yet, of any department of medicine; but ours is still too young, too immature, to have nearly reached the symmetry and stature which the rich materials, ready and offering themselves for our use, are waiting to bestow upon it. This way lies your path; these fields are ripe for your reaping; their harvest is for your gathering, winnowing and storing, in the seminaries of our science.

The systematic principles of the parent profession, and its remedial means and methods, all have references available and essential to our art; but it rests upon us to give them adjustment to our own uses. The teachers of medicine and surgery know nothing of dentistry, and are utterly incapable of providing for its growth, or directing the manner of its development. Their science abounds with material for the edification of ours; but that law which rules the assimilative processes of the living body, obtains with like force in the several professions concerned with its constitution and charged with its health. Each member, each separate organ, appreciates and appropriates, from the common vital current that flows through them all, those elements of life and growth which it specially requires. The figure, which is also, in the strictest sense, the fact, teaches you that *you* must select, secrete and elaborate your own vitality from the current life-blood of the general healing art.

In the exercises of your society, its essays, reports and debates, room and requirement will be present for turning all the streams of medical and surgical science into the channel of our progress. One in physiology, another in histological anatomy, a third in the principles of surgery, a fourth in pathology, a fifth in materia medica, a sixth in practice, prophylactic and therapeutic, a seventh in chemistry as applied to the art—each will bring its contribution—each his revelation, while others will interpret, and all will profit in their special provinces of practice and speculation; and thus, in the happily descriptive language of the Apostle to the Ephesians, which has all the exactitude of a scientific statement of the vital functions of circulation and assimilation, "the whole body fitly joined together and compacted by that which every joint supplieth, according to the effectual working in the measure of every part, maketh increase of the body unto the edifying of itself in love."

But when all the branches of technical medicine are thus explored and their contributions received, the relations and dependencies of your art are not yet all included. Natural history and comparative anatomy are rich in material and abundant in suggestion for your service, as a glance at Agassiz' Principles of Zoology, or the Odontography of Owen, the former in a small popular treatise, the last in two volumes, with 168 plates, (exclusively of the teeth of the vertebrate animals,) will abundantly demonstrate.

Metallurgic and mechanical science are mines of wealth to the dentist, to be smelted and moulded into useful application, by the labors of those who have the special qualification, taste and favoring conditions for the work; and Mechanism and Manipulation open spheres of discovery and improvement for other useful modifications of individual talent. And finally, for I must close this rapid and sketchy enumeration; Esthetical principles and art have a commanding worth among the researches that invite your zealous devotion; and Physiognomy, a mere play of curious speculation to the disciples of other spheres of useful learning, becomes a practical requirement of yours.

If the Painter is concerned with color, light and shade, and their correspondences; and the Statuary, with form and symmetry, and their significance, you, as much as either, or both, require the nicest perceptions and exactest adjustments of these elements of expression in the perfection of your work.

In all these varied departments of science—in all this large range of practical and imitative art, dentistry is deeply involved, and it is in the programme of your duties to develop all they have of use and beauty, and incorporate it into the system and service of our art.

The necessity for a wide distribution of functions, and a close combination of effort, needs no argument after a survey of the multiform requirements that are to be met. The interlinking and dependency, the reciprocal exchange of service, and the mutuality of stimulation to exertion, which your organization provides for, stands clearly demonstrated in the light of a fair apprehension of our profession's conditions, demands and destiny.

By all that your predecessors have achieved, which is at once your inheritance and debt—by all the toils and hopes and triumphs embodied in the progress that has been attained—by all the prophecies springing from the success realized—by all the trust implied in the stewardship committed to your hands—by the hopefulness of your own ambition, and the duties that you owe to your brethren and the world—you are commissioned and commanded to a worthy performance of your work.

Your diplomas do not mean that your studies are finished and your attainments completed; your preceptors do not dream that their teachings reach the natural limits, or cover, or complete, the boundaries of our broad field of professional learning. The past was theirs, but the future is yours, and we look to you to make that future worthy of that past, by the distance in excellence which your achievements shall put betwen them.

But your commitments and obligations follow you from your conventional and fraternal intercourse with each other into the relations of private professional life, and the same spirit requires conformable conduct there. The recruits, series after series, that are to be your compatriots first, and afterwards your successors, come fairly within your responsibilities. The system of private tuition which will be largely in your hands or otherwise under your influence, is one of the weightiest duties, and one of the worthiest, you are about to assume. Look well to the preparatory education, the personal qualities, the morals and the habits, of those whom you admit to the opportunities of your tutorship. And then, under the most solemn obligations of honor and conscience, and with the strictest fidelity to them and to the profession, do your whole duty to them. See that their studies are systematic, liberal and thorough. Let neither the interests of business, nor the selfishness or indolence that tempt, nor the difficulties that embarrass, hinder you from preparing them for the best reception of their subsequent collegiate instruction. The utmost fidelity of the colleges in the discharge of their functions will fail of its highest aims, if you send them unworthy, incapable, or undisciplined subjects for their teachings. Allow me to say, moreover, that a general elevation and improvement of the system of private study will re-act with prodigious force upon the Faculties of our colleges, and push them also up toward a higher grade and style of education; not only by the relief it will afford them from the labor of horn-book instruction, but by the force of that criticism with which it will qualify their classes. Bear constantly in mind that you are the educators of the future profession in as absolute, if less obvious degree, as those who put their names to your diplomas. Out of the faithful exercise of this power which is in your hands, we trust, will speedily arise a greatly enhanced requirement in the educational customs of the country, and under its influence, quackery will rapidly go down, and the usefulness and honor of the profession as rapidly rise to their natural rank in liberal learning.

What more shall I add? how else or better can I enforce the just requisitions of the profession, of the world, and of the future upon you? The topics are not exhausted, for they are exhaustless, nor the solicitudes satisfied, for they are importunate, and without limit, as their objects are lofty and holy.

But your own ambition, your attitude and commitments, are a sufficient assurance. You have taken your advanced position resolutely. In the spirit of professional chivalry you have taken upon you the high adventure, and will not fail of the achievement.

We commend and commit you to your task, and, with our heartiest and hopefullest words of cheer, trust you for the issue

